# Automated Tracking of Biopolymer Growth and Network Deformation with TSOAX

**DOI:** 10.1038/s41598-018-37182-6

**Published:** 2019-02-08

**Authors:** Ting Xu, Christos Langouras, Maral Adeli Koudehi, Bart E. Vos, Ning Wang, Gijsje H. Koenderink, Xiaolei Huang, Dimitrios Vavylonis

**Affiliations:** 1 Department of Computer Science and Engineering, Lehigh University, Bethlehem, PA 18015 USA; 2 Department of Physics, Lehigh University, Bethlehem, PA 18015 USA; 3 AMOLF, Living Matter Department, 1098 XG Amsterdam, The Netherlands; 4 Department of Molecular Genetics, The Ohio State University, Columbus, OH 43210 USA; 5 Present Address: Howard Hughes Medical Institute and Department of Cell Biology, Harvard Medical School, Boston, MA 02115 USA; 6 Present Address: College of Information Sciences and Technology, Penn State University, University Park, PA 16802 USA

## Abstract

Studies of how individual semi-flexible biopolymers and their network assemblies change over time reveal dynamical and mechanical properties important to the understanding of their function in tissues and living cells. Automatic tracking of biopolymer networks from fluorescence microscopy time-lapse sequences facilitates such quantitative studies. We present an open source software tool that combines a global and local correspondence algorithm to track biopolymer networks in 2D and 3D, using stretching open active contours. We demonstrate its application in fully automated tracking of elongating and intersecting actin filaments, detection of loop formation and constriction of tilted contractile rings in live cells, and tracking of network deformation under shear deformation.

## Introduction

Microscopic studies of biopolymer networks reveal dynamical and mechanical properties important for their function in tissues and living cells. Quantitative studies have been advanced by using *in vitro* models, where biopolymers such as actin filaments, intermediate filaments and microtubules are purified and reconstituted into networks with well-controlled conditions. Extracting quantitative information from such studies is challenging due to low image signal to noise ratio (SNR), and the large size of the data that requires automated extraction. Recently, methods and software have been developed for extraction of network structure in biopolymer images of low SNR for a single time point in 2D and 3D^1–5^. For example, the SOAX software which is based in stretching active contours^1^, uses a curvilinear network of multiple open curves (“snakes”) to automatically extract 2D and 3D networks. Very little work however exists to quantify the evolution of network structures in 2D/3D time-lapse images. Some previous efforts focused on tracking of individual filaments^6–8^ or filament tips^9^.

Automated tracking of dynamic biopolymer networks is challenging because of the variability of filament dynamics, which can include filament growth and shrinkage, filament or network node appearance and disappearance, and global or local network deformation. To study how the network remodels and deforms in time, the goal of tracking is to generate a track for each filament, segment or node in the network. Examples of studies that can benefit from automated network tracking include studies measuring the elongation kinetics of individual actin or microtubule filaments that intersect with one another as they polymerize^10^, measuring the deformation of cross-linked fiber networks under external forces, which relates to their mechanical properties^11,12^, as well as network structures that form in cells through polymerization and depolymerization^13^.

**Figure 1:**
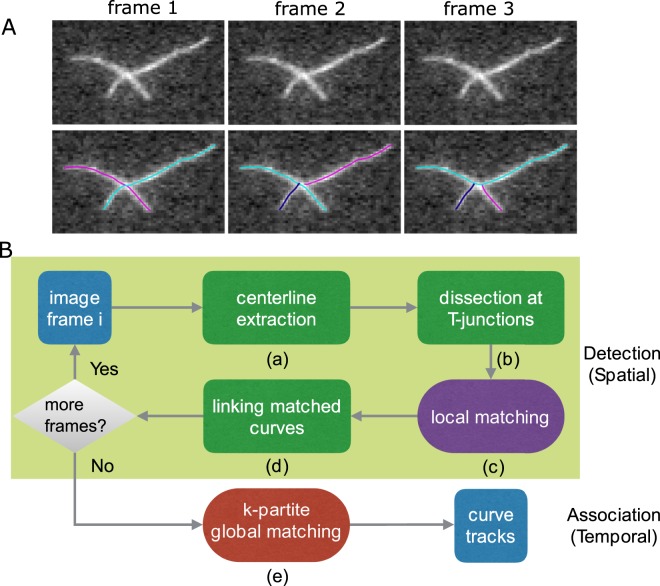
Network tracking algorithm. (**A**) Example of time lapse sequence (three identical frames, for simplicity). Image of actin filaments from Fujiwara *et al*.^10^ 1 pixel = 0.17 *μ*m. After filament segment detection in each separate frame (shown with different colors), each frame may be extracted by a set of curves with different topology. Because of the different detected structures across frames, the network can not be tracked well using only a global matching algorithm. We thus added a local matching procedure to maintain consistent network topology. (**B**) Flowchart of the method consisting of a detection phase (shaded region) and a matching (i.e. temporal association) phase. The output is a set of “curve tracks,” one for each filament/segment in the network. The dark green steps are the same as in the method used in SOAX^1,19^. The steps in the other boxes are new and described in this work.

Formulating multi-frame object tracking as finding the minimum or maximum path cover in a *k*-partite graph has been successful for tracking cells^14^, tips of microtubules^15^, and point features in natural images^16^. Beside low computation complexity, the major benefit of this formulation is that it allows for false negative/positive classification of detection results (that aids when detection is influenced by image noise), and appearing/disappearing/reappearing of tracked objects. However, direct application on tracking networks of filaments does not work well because temporal consistency of network topology is not considered (see Fig. 1A). Another complication is that detection error can propagate to the correspondence phase (i.e. the process assignment of detected filaments to tracks) thus adversely affecting tracking performance. Using temporal information in the detection step has been useful in cell tracking^17^. The method in^18^ improved reconstruction of tree structures by incorporating temporal information when building a spatio-temporal graph of seed points with high tubularity. No temporal correspondence was generated for individual tree branches however.

We integrated the approaches described above into a novel approach for biopolymer network tracking that (i) extends the global *k*-partite matching method to deal with curved filaments, and (ii) implements a local matching procedure during the detection phase to improve the accuracy and consistency of network topology across frames. This method has been implemented in an open source software tool, TSOAX (Trackable Stretching Open Active Contours), which is an extension of SOAX (designed for static 2D/3D images) to time-lapse movies of 2D/3D images.

## Materials and Methods

### Outline of Computational Method

The method used in TSOAX is divided into a detection phase and a matching phase. In the detection phase corresponding to the shaded region in Fig. 1B, the centerlines of a network are extracted frame by frame using multiple open curves^1,19^ (Fig. 1B(a)), which are automatically initialized and elongated sequentially. They stop elongation upon reaching filament tips or colliding with other converged curves. These curves are dissected at colliding points (“T-junctions”) which are clustered into detected network junctions (Fig. 1B(b)). This procedure uses the same method and parameter values as in the SOAX software for single frame network detection^1^. In order to produce temporally consistent network topology for the matching phase, we introduce a temporal information based local matching step (Fig. 1B(c)). Matched curves are linked together as the detection result for a filament (Fig. 1B(d)). After all the frames are processed, temporal correspondence among all the curves in the sequence are found by a global *k*-partite graph matching framework (Fig. 1B(e)).

Readers primarily interested in the software and its application to specific examples can skip directly to the Software Implemention section and the Results section.

### *k*-Partite Global Matching of Multiple Curves

To generate tracks from the extracted curves such that they correspond to the same structures in an evolving network, we extend the *k*-partite graph matching framework to the problem of finding temporal correspondence of multiple curves in *k* frames (Fig. 1B(e)). We construct a *k*-partite acyclic directed graph *G* = (*V*, *E*), where the vertex set *V* represents the set of extracted curves on all frames (each vertex being a curve) and the edge set *E* represents temporal links (i.e. “potential” matching) among curves. Specifically, a directed edge $({v}_{i}^{l},{v}_{j}^{m})\in E$ links the *i*th curve in frame *l* to the *j*th curve in frame *m* if *l* < *m*, i.e., an edge can only point forward in time (also curves from the same frame cannot be associated with one another). A path in *G* is an ordered sequence of vertices and their directed edges in between. A path cover of *G* is a set of paths such that each vertex belongs to exactly one path, which implies each curve is associated exactly once temporally.

Since we would like curves with similar position and geometry to belong to the same track, we define below an edge weight $w({v}_{i}^{l},{v}_{j}^{m})$ to quantify the dissimilarity between the two curves. Then we define a set of tracks to be the path cover C* that minimizes the total dissimilarity of all the paths among all possible path covers of *G*:

1 $${C}^{\ast }(G)=\mathop{{\rm{argmin}}}\limits_{C}\sum _{p\in C}\sum _{({v}_{i}^{l},{v}_{j}^{m})\in p}w({v}_{i}^{l},{v}_{j}^{m}),$$

where *p* is a directed path in a path cover *C*.

The weight of an edge $w({v}_{i}^{l},{v}_{j}^{m})$ encodes the difference in location and geometry between curves ${v}_{i}^{l}$ and ${v}_{j}^{m}$. Given that ${v}_{i}^{l}$ and ${v}_{j}^{m}$ are represented by a linear sequence of snake points, we define the average Euclidean distance between them to be

2 $$d({v}_{i}^{l},{v}_{j}^{m})=\frac{1}{\mathrm{2|}{v}_{i}^{l}|}\sum _{{x}\in {v}_{i}^{l}}\mathop{{\rm{\min }}}\limits_{{\bf{y}}\in {v}_{j}^{m}}\Vert {x}-{y}\Vert +\frac{1}{\mathrm{2|}{v}_{j}^{m}|}\sum _{{\bf{y}}\in {v}_{j}^{m}}\mathop{{\rm{\min }}}\limits_{{x}\in {v}_{i}^{l}}\Vert {y}-{x}\Vert ,$$

where x and y are points in ${v}_{i}^{l}$ and ${v}_{j}^{m}$, respectively. Here, $|{v}_{i}^{l}|$ and $|{v}_{j}^{m}|$ are the number of snake points in the corresponding curves. The weight is computed as:

3 $$w({v}_{i}^{l},{v}_{j}^{m})=\{\begin{array}{cc}{e}^{c(m-l-\mathrm{1)}}d({v}_{i}^{l},{v}_{j}^{m}), & {\rm{if}}\,l < m\,{\rm{and}}\,{\rm{value}}\,{\rm{smaller}}\,{\rm{than}}\,\eta \\ \eta , & {\rm{otherwise}}\end{array}$$

where *c* is a factor controlling how much curves from different frames are penalized. A maximum weight *η* is assigned to curves that are so far apart from one another (in space or frames) that there is no need to distinguish among them. This maximum value should be at least several pixels so in the examples in this paper we set *η* = 20 pixels.

Solving Eq. 1 generates a set of tracks, each containing a sequence of curves. The curves of a track are not necessarily from consecutive frames since a structure can be missing for several frames and reappearing again. A track can be zero-length, containing a single vertex that corresponds to one curve with no predecessor or successor in time.

The minimum path cover of *k*-partite graph can be solved in polynomial time by transforming it into bipartite matching. We construct a complete bipartite graph *B* = (*V*′, *E*′) from *G* = (*V*, *E*) as follows. The two partite vertices *V*_+_′ and *V*_−_′ of *B* are copies of *V*, i.e. *V*′ = *V*_+_′ ∪ *V*_−_′ where *V*_+_′ = *V*_−_′ = *V*. For each edge $({v}_{i}^{l},{v}_{j}^{m})\in E,\text{with}\,l < m$, we copy its weight to edge $({v}_{i+}^{l}\text{'},{v}_{j-}^{m}\text{'})\in E^{\prime}$. All remaining edges that correspond to matching within the same frame or backward matching are assigned the maximum weight *η* (Eq. 3). It has been proved that the minimum path cover of *G* in Eq. 1 corresponds to the minimum matching of the bipartite graph *B*^16^. By converting Eq. 1 to the problem of minimum matching of bipartite graph *B*, we can find the best path cover using well-established algorithms such as the Hungarian or Hopcroft-Karp algorithms.

### Enforcing Temporal Consistency of Network Topology by Local Matching

One limitation of the above global matching is that it depends on a consistent detection result, i.e., the way extracted curves “partition” a network stays the same across frames. However, this temporal consistency is not enforced in the network detection phase. As in the situation shown in Fig. 1A, the accuracy of global matching will decrease when the extracted network topology does not have temporal consistency. Therefore we introduce a novel local matching step (Fig. 1B(c)) where dissected curves are linked together respecting both local cue and temporal information from previous frames, in order to enforce a “consistent partition” of the network over time. Specifically, two dissected curves incident at a junction should have higher affinity (more likely to be linked) if 1) they form a smooth curve passing through the junction if linked; 2) their changes compared to previous frames are similar. This topological complexity is usually not present in the task of tracking point objects such as cells^14,20^, tips of microtubules^15^, or other point features in natural images^16^.

As described above, the curve segments arriving at a junction are dissected and the goal is to connect pairs of these curves segments into a combined curve. We model this local matching between curves as a bipartite matching (as in the global matching phase during track generation, and performed before the global matching) by trying to find the matching that minimizes a dissimilarity measure. We construct a complete bipartite graph containing two copies of curve tips incident at a network junction. The edge weights are the pairwise dissimilarity $D({{x}}_{i}^{l},{{x}}_{j}^{l})={D}_{ori}({{x}}_{i}^{l},{{x}}_{j}^{l})+{D}_{tem}({{x}}_{i}^{l},{{x}}_{j}^{l})$ between tip ${{x}}_{i}^{l}$ and ${{x}}_{j}^{l}$ of the dissected curve ${u}_{i}^{l}$ and ${u}_{j}^{l}$ in frame *l*, respectively.

The first term *D*_*ori*_ encodes the difference of tangential orientation at tips based on the current frame,

4 $${D}_{ori}({{x}}_{i}^{l},{{x}}_{j}^{l})=\{\begin{array}{cc}\mathrm{1,} & {\rm{if}}\,{u}_{i}^{l}={u}_{j}^{l}\\ 1-\theta /\pi , & {\rm{if}}\,{u}_{i}^{l}\ne {u}_{j}^{l}\end{array}$$

where *θ* ∈ [0, *π*] is the angle between the tangents at the curve tips ${{x}}_{i}^{l}$ and ${{x}}_{j}^{l}$. When two curves face in opposite directions towards each other at a junction, *θ* is close to *π* and the dissimilarity is low.

The second term *D*_*tem*_, utilizing temporal information, encodes the difference of the distance to the curves in the previous frame that have been already locally matched. We define ${v}_{i}^{l-1}$ to be the closest (already locally matched) curve for ${u}_{i}^{l}$ in frame *l* − 1

5 $${v}_{i}^{l-1}=\mathop{{\rm{\arg }}\,{\rm{\min }}}\limits_{r\in {V}^{l-1}}f({u}_{i}^{l},r),$$

where *V*^*l*−1^ is the set of detected curves in the previous frame. The measure $f({u}_{i}^{l},r)$ is the one-way distance between curves ${u}_{i}^{l}$ and *r*, taking into consideration both physical proximity and orientation

6 $$f({u}_{i}^{l},r)=\frac{1}{|{u}_{i}^{l}|}\sum _{{\bf{y}}\in {u}_{i}^{l}}(\mathop{{\rm{\min }}}\limits_{{\bf{z}}\in r}\Vert {y}-{z}\Vert ){e}^{-|cos\varphi (y,z)|}\mathrm{.}$$

Here *ϕ*(*y*, *z*) is the angle between the tangent at point *y* of curve ${u}_{i}^{l}$ and the tangent at the point *z* of *r* that is closest to point *y*. We define

7 $${D}_{tem}({{x}}_{i}^{l},{{x}}_{j}^{l})=\{\begin{array}{lc}\mathrm{1,} & {\rm{if}}\,{v}_{i}^{l-1}\ne {v}_{j}^{l-1}\\ |\,f({u}_{i}^{l},{v}_{i}^{l-1})-f({u}_{j}^{l},{v}_{j}^{l-1})|, & {\rm{if}}\,{v}_{i}^{l-1}={v}_{j}^{l-1}\end{array}$$

Thus, if two dissected curves are close to different curves of the preceding frame, their dissimilarity is given the maximum value 1. If they are close to the same curve of the preceding frame, the dissimilarity is decreasing depending on how close enough, in terms of position and orientation, each curve is to their common closest curve. The reason of using a difference of *f* in Eq. 7 instead of requiring that both are small is that we found this to better account for cases where there was an overall shift of the curve.

As an example of what the local matching method achieves, consider a filament *v* elongating and passing another filament *u*, creating a junction point at frame *l*_0_. A curve *u*^*l*^ will be detected along *u* in frames prior to the formation of the junction (*l* < *l*_0_). The curve segments at the junction point at frame *l*_0_ that are close to ${u}^{{l}_{0}-1}$ will be spliced together because of their low value of *D*_*tem*_ and low value of *D*_*ori*_. The segments of curve *v* on either side of the junction with *u* will be spliced together because of the low value of *D*_*ori*_. The resulting topology would thus be consistent over time.

### Software Implementation

**Figure 2:**
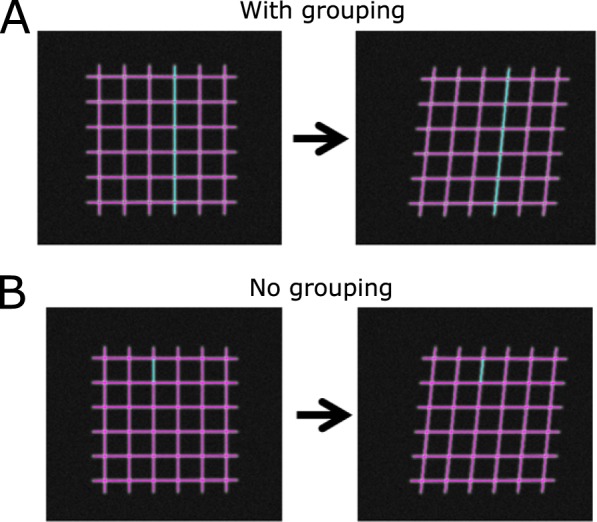
Grouping option in TSOAX. (**A**) The algorithm applied to track fibers that extend beyond junctions. (**B**) Tracking of segments of fibers connecting network junction points.

**Figure 3:**
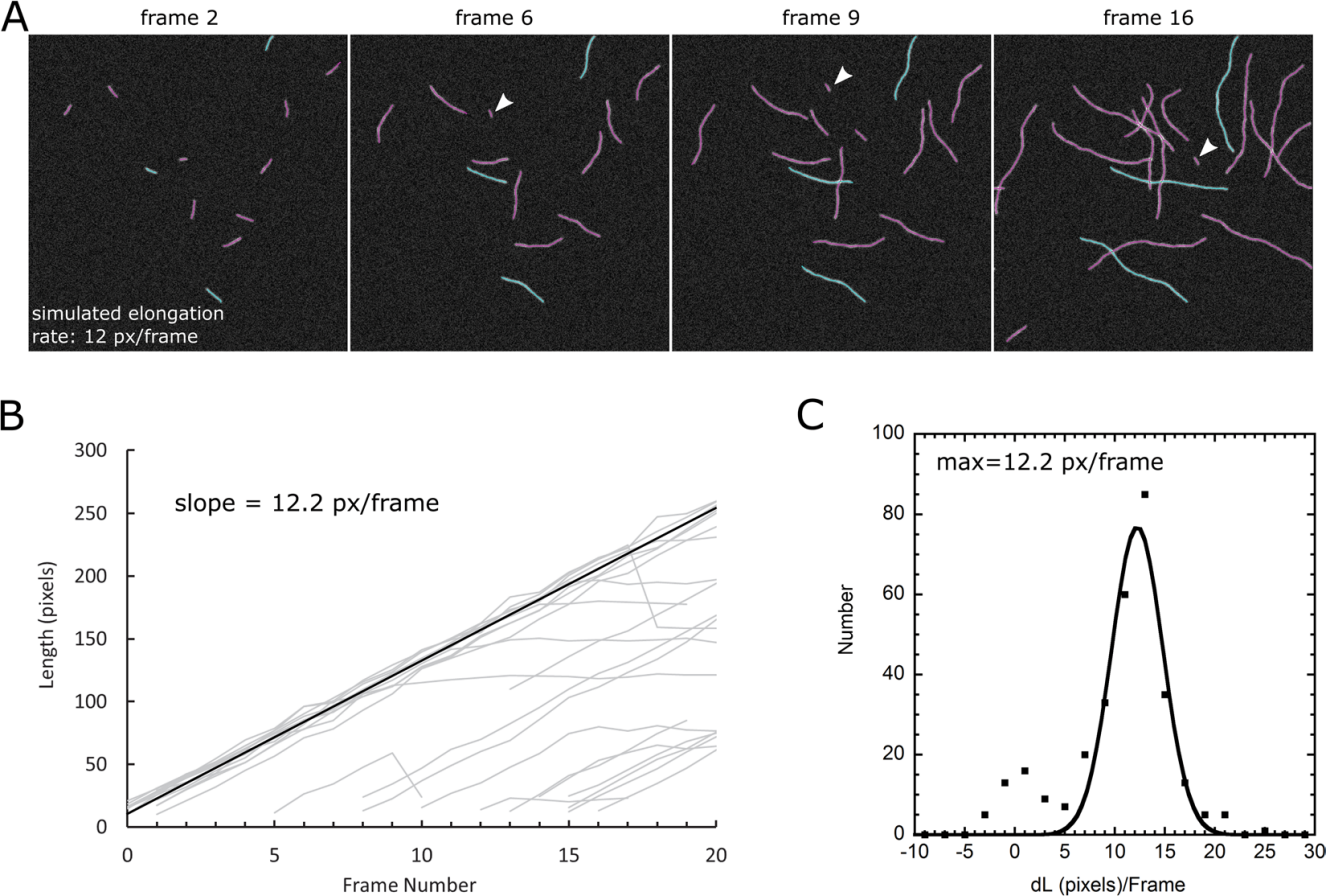
Tracking actin filaments in simulated TIRFM images. (**A**) Detected filaments in 2D simulated images. Arrow heads: new filaments. Blue curves show snakes belonging to the same track (three tracks shown). (**B**) Plot of length versus time for all tracks detected by SOAX. (**C**) Histogram of snake length change per frame and Gaussian fit after filtering out the data in the small peak around *dL* = 0.

**Figure 4:**
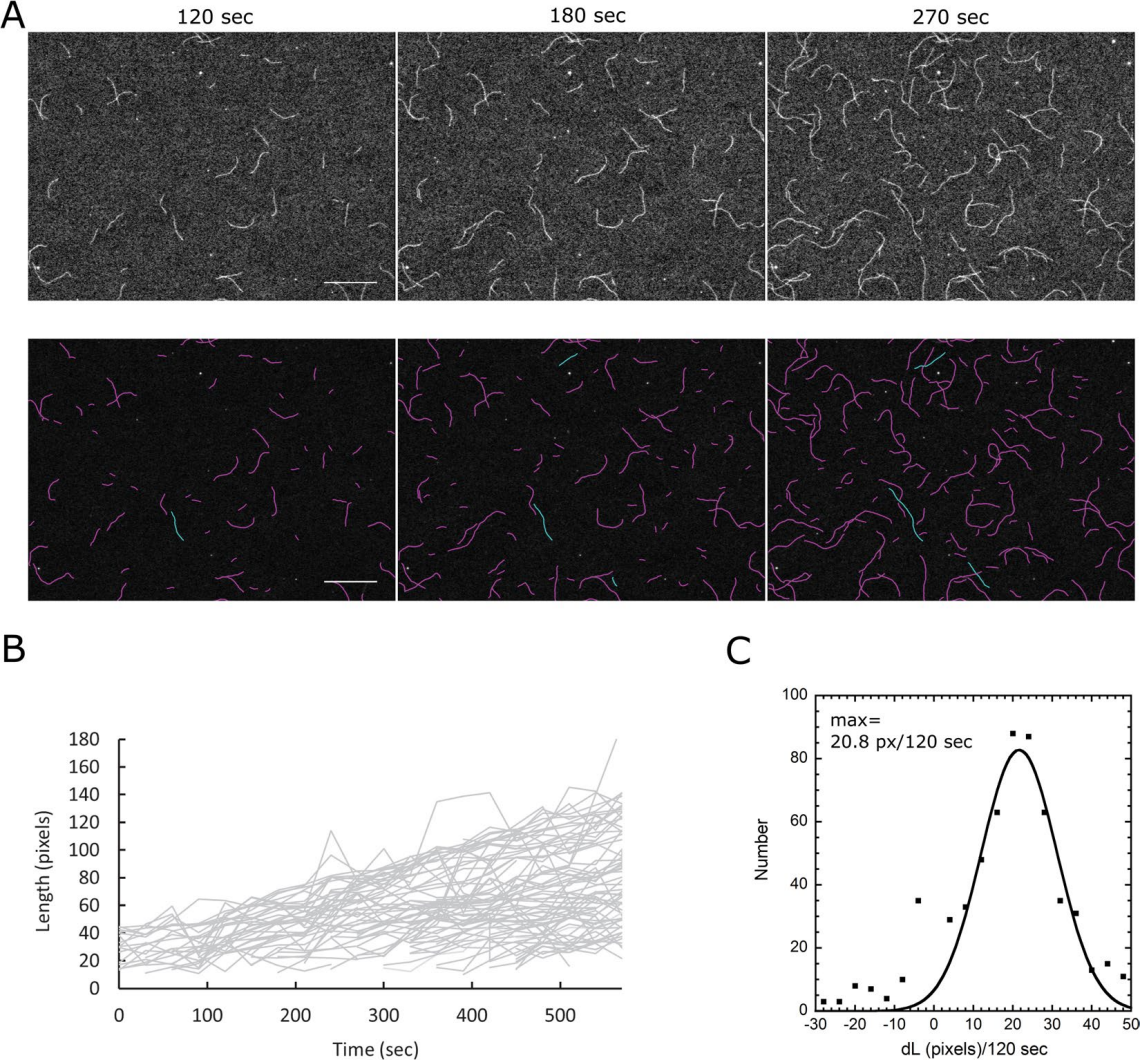
Tracking actin filaments in a TIRFM image sequence from Fujiwara *et al*.^10^. (**A**) First row: sample raw images. Bottom row: Tracked filaments. Blue curves show snakes belonging to the same track (three tracks shown). The pixel size is 0.17 *μ*m and the time interval between successive frames 30 sec. Bar: 20 *μ*m. (**B**) Plot of length versus time for all tracks detected by TSOAX. (**C**) Histogram of snake length change per frame and Gaussian fit after filtering out the data in the small peak around *dL* = 0. Given 370 actin subunits per *μ*m along an actin filament and 0.17 *μ*m per pixel, the peak at 20.8/120 = 0.173 pixels/sec corresponds to 10.9 subunits/sec.

**Figure 5:**
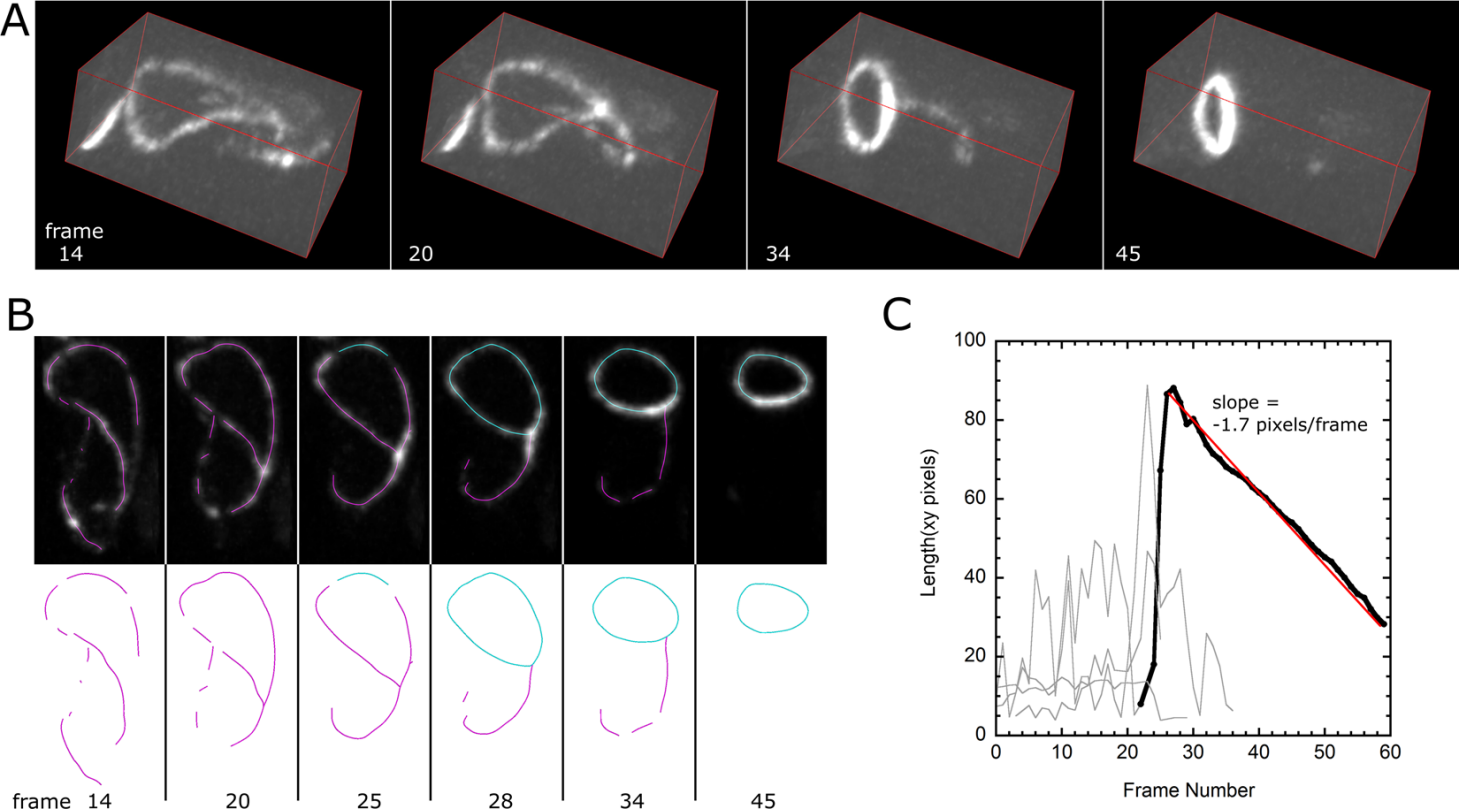
Tracking of ring constriction in *mid1Δ* fission yeast cells expressing myosin light chain Rlc1-GFP. (**A**) Time-lapse confocal microscopy image of cells assembling a ring from Rlc1 segments distributed along the cell cortex. The cell shape is tubular and has diameter ∼4 *μ*m, with its long axis along the long axis of the box. Image size: 36 × 85 pixels with 1 *μ*m = 6.95 pixels. A 3D stack of 27 *z*-slices separated by 0.3 *μ*m was obtained every 1 min. (**B**) Side view showing detected snakes. Highlighted in blue is the track of the snake corresponding to the constricting ring. (**C**) Plot of snake segment length versus time for all tracks allows identification of the time point of ring detection and measurement of constant constriction rate 0.24 *μ*m/min (red line).

**Figure 6:**
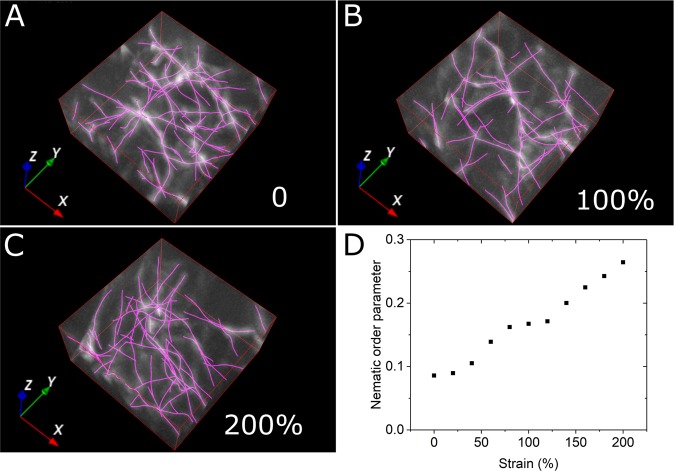
Measuring deformation of 3D fibrin networks in confocal rheology image sequence. The images in panels (A–C) show a small region of a sample of fluorescently labeled fibrin (2 mg/ml). The sample underwent sequential mechanical deformation along the *x* direction by an externally applied shear in 10% intervals, followed by imaging at each interval. Panels (A–C) show images at 0, 100% and 200% strain and extracted network (with no grouping). The dimensions of the image along a confocal size is 16 × 16 *μ*m (100 × 100 pixels). There are 50 *z* slices, separated by 0.16 *μ*m. (**D**) Graph shows the orientation-independent nematic order parameter as a function of applied strain, combining the tracked filaments from four different images (of the same size as the example in this figure) to calculate one single order parameter per strain level.

**Figure 7:**
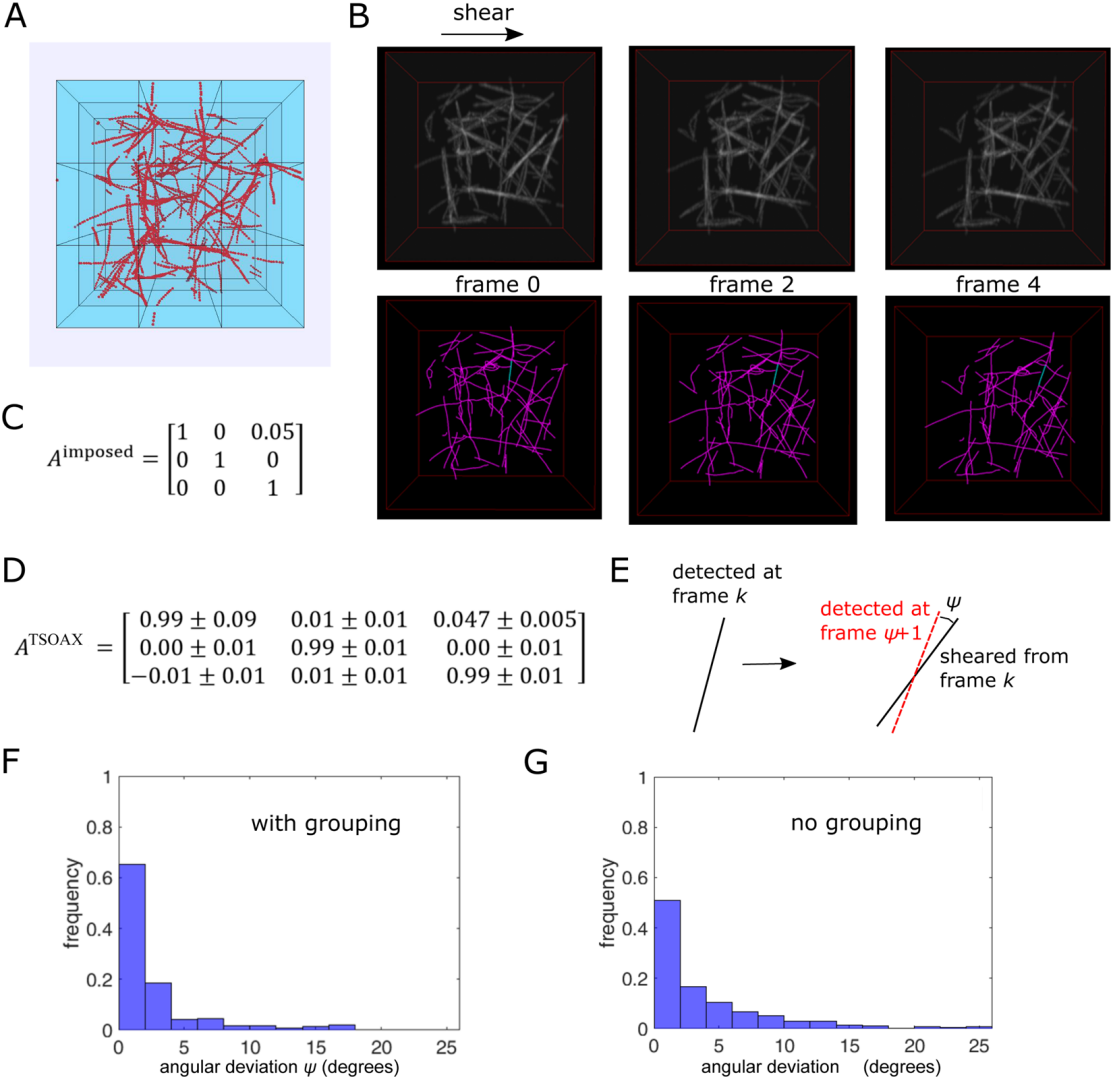
Tracking shear deformation of simulated 3D network. (**A**) Snapshot of Brownian dynamics simulation of actin filaments forming a network of bundles and single filaments. Simulations correspond to 3.2 *μM* actin: 120 filaments of length 1.1 *μm* in box of side 3 *μm* (whole blue box). (**B**) Top row: synthetic image (200 pixels/side) generated from filament coordinates in panel A under four successive shear transformations. Bottom row: TSOAX results (with no grouping) with example of tracked segment in blue. (**C**) Shear matrix per frame. (**D**) Transformation matrix calculated from TSOAX tracked snakes with grouping between frame 0 and 1 using 30 samples (10 triplets) out of 55 tracked snakes (mean ± standard error). (**E**) Definition of angular deviation between axis of detected snake and axis of sheared snake of preceding frame of same snake track. (**F**) Distribution of angular deviation between the snakes of all successive frames of a sequence of four frames (with grouping, *n* = 237). (**G**) Same as F but with no grouping (*n* = 447).

**Figure 8:**
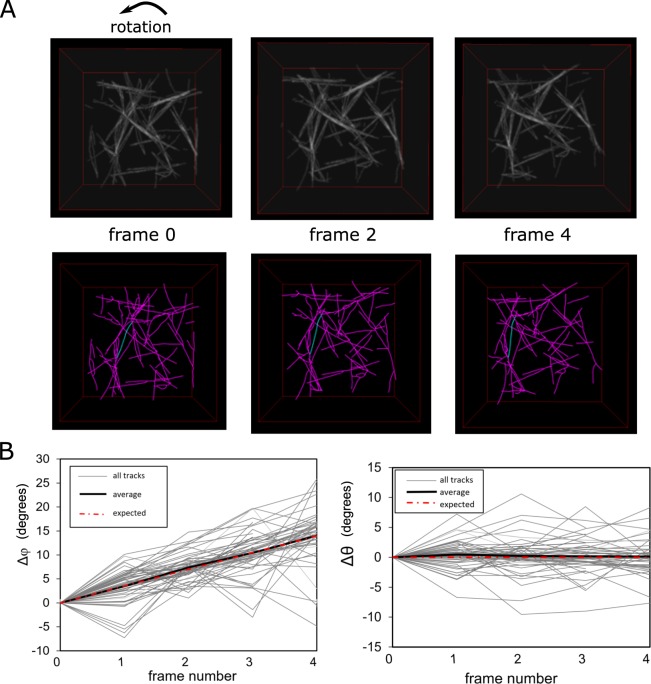
Tracking uniform rotation of simulated 3D network. (**A**) Top row: synthetic image (200 pixels/side) generated from filament coordinates in panel A of Fig. 7A (top view). The filaments are rotated by 3.5 degrees along the *z* axis in a total of four iterations. Bottom row: TSOAX results (with no grouping) with example of tracked segment in blue. (**C**) Change of azimuthal (*ϕ*) and polar (*θ*) angle per frame vs frame number with grouping for 53 snakes presents in more than four frames.

We implemented the above methods into the TSOAX software (http://athena.physics.lehigh.edu/tsoax), built as an extension of SOAX for static images (http://athena.physics.lehigh.edu/soax). The parameters for filament detection in each time frame are the same in both programs. Two additional parameters in TSOAX are: (1) Parameter *c* controlling the weight by which snakes detected at nearby locations in space are assigned to the same track, as a function of the number of time frames separating them (see Eq. 3). The value of 1/*c* is the number of frames beyond which the probability of assigning snakes to the same track decreases exponentially with frame number separation. This parameter should be increased (up to a value of order *c* = 1/frame) to improve track continuity over successive frames. (2) Option “Grouping,” which enables the grouping process of snakes at detected junctions prior to tracking (Fig. 2). This option can be enabled for tracking of intersecting elongating filaments (Figs 3–5) and disabled for tracking the movement of filament segments in between junction points (Figs 6–8).

### Confocal Rheology of Fibrin Networks

Human plasma fibrinogen (Plasminogen, von Willebrand Factor and Fibronectin depleted) and human *α*-thrombin were obtained from Enzyme Research Laboratories (Swansea, United Kingdom). All chemicals were obtained from Sigma Aldrich (Zwijndrecht, The Netherlands). Fibrinogen was dialysed against buffer containing 20 mM HEPES and 150 mM NaCl at a pH of 7.4, and mixed with 500 mM CaCl _2_ at room temperature to a final assembly buffer containing 20 mM HEPES, 150 mM NaCl and 5 mM CaCl _2_. For fluorescence microscopy, fibrinogen labeled with Alexa Fluor 488 (Life Technologies, Bleiswijk, The Netherlands) was mixed with unlabeled fibrinogen in a 1:19 molar ratio.

Assembly was initiated by the addition and quick mixing of 0.5 U/ml of thrombin, after which the sample was quickly transferred to the confocal rheometer. Evaporation of solvent during assembly was prevented by adding a layer of low-viscosity mineral oil (M3516, Sigma Aldrich) on the liquid-air interface.

Confocal microscopy in combination with shear rheology was performed on a home-built setup, consisting of an Anton Paar rheometer head (DSR 301, Graz, Austria) placed on top of an inverted microscope equipped with a Yokogawa CSU-22 spinning disk confocal head, a Hamamatsu EM-CCD C9100 Digital Camera, and a 100x oil-immersion objective. The bottom plate consisted of a microscopy coverslip, while the top plate was a 20 mm stainless steel plate. Imaging was performed half-way the centre and the edge of the top plate. After polymerization, instantaneous strain steps of 10% were applied. Every strain step was held for 1 minute during which three-dimensional stacks were taken, starting 10 *μ*m above the glass interface. The stacks consisted of 50 steps in *z* with a spacing of 0.16 *μm* and an exposure of 0.5 s per frame.

### Synthetic Images of Elongating Filaments

Images of elongating 2D filaments that resemble actin were generated using a walk of constant step size *δ* = 0.05 pixels. An angle *θ* between the displacement vectors of successive steps was chosen from a Gaussian distribution centered at 0 and standard deviation *σ*_*θ*_ = 0.015 radians. This generates a polymer with persistence length ${l}_{p}\,=\,\delta /{\sigma }_{\theta }^{2}\,=\,222$ pixels^8^. For a typical actin filament persistence length of 17 *μ*m, this corresponds to pixel size of 76 nm. The filaments were elongated at 12 pixels per frame by adding, on average, 240 segments of length *δ* per frame (picked from 300 attempts to add a segment and accepting each with probability 0.8 to generate fluctuations in elongation with standard deviation 1.5 pixel per frame). Each segment of the walk was assigned a brightness of 5 units that fluctuated within 10% of the average value, to simulate intensity fluctuations along the filament. Images were generated by convoluting the points representing the trajectory of the walk with a Gaussian kernel of standard deviation 1.5 pixels in the *x* and *y* direction. A random number picked from a uniform distribution between 0 and 10 was added at each pixel to simulate background camera noise.

### Synthetic Images of Sheared and Rotated Polymer Network

We created synthetic movies of sheared and rotated polymer networks starting from a Brownian dynamics simulation of attractive semiflexible polymers (Fig. 7A). We used the method of Tang *et al*.^21,22^ to generate polymerizing semiflexible actin filaments in a 3D volume with periodic boundary conditions. Actin filaments were simulated using a coarse-grained bead-spring model in which each bead represents a segment of the actin filament of length *l*_0_, corresponding to 14 actin subunits. Polymerization of actin filaments was simulated as elongation of the first segment of this semi-flexible polymer with rate *k*_+_*c*_*actin*_(*t*), where *k*_+_ is the polymerization rate constant and *c*_*actin*_(*t*) is the bulk monomer concentration at time *t*. As soon as the segment reached twice *l*_0_, a new bead was introduced to the polymer chain. Assuming an initial concentration of unpolymerized actin monomers ${c}_{actin}^{0}$ and an initial number of actin filament seeds *N*_*fil*_, the concentration of bulk actin, *c*_*actin*_(*t*), was reduced over time to represent the calculated amount of remaining bulk actin monomers. The attractive interaction between actin filaments was simulated as a short range harmonic attractive force with range *r*_*atr*_, spring constant *k*_*atr*_ and equilibrium length *r*_0_ as in Tang *et al*.^21^. Using this set up and parameters values in Table 1, we performed a 3D simulations for 4 s, over which filaments reached a length of 1.1 *μm* (Fig. 7A). The diffusion and attraction between filament beads led to the formation of a bundle network^21^. The 4 s snapshot to the network (a transient structure in the simulations) was used to generate an example of a 3D static network image.

**Table 1:** Reference Parameter Values Used in Simulations to Generate Sheared and Rotated Networks.

Parameter	Description	value
*L*_*box*_	Domain size	3.0 *μm*
${c}_{actin}^{0}$	Initial actin concentration	3.2 *μM*
*N*_*fil*_	Number of filaments	120
*T*	Temperature	300 K
*l*_*p*_	Persistence length	17 *μm*
*η*	Viscosity (350 times larger than water)	0.301 *pNs*/(*μm*^2^)
*k*_+_	Polymerization rate constant	10 (*μMs*)^−1^
*l*_0_	Filament segment length	0.037 *μm*
*k*	Spring constant between filament beads along a filament	100 *pN*/*μm*
*k*_*atr*_	Attraction force spring constant	0.5 *pN*/*μm*
*r*_0_	Attraction force equilibrium length	0.012 *μm*
*r*_*atr*_	Attraction force range	0.15 *μm*

We then applied a simulated Gaussian microscope point spread function to a snapshot of the simulation and added noise to create a 3D synthetic image stack (Fig. 7B), similar to the synthetic images of Fig. S1 in Bidone *et al*.^22^. Images of a sheared network were generated by applying successive shear transformations to the coordinates of each bead of the filament in the simulation snapshot, without applying any other movement or deformation to the filaments. The transformation of each bead from one frame to the next is

8 $$[\begin{array}{c}x^{\prime} \\ y\text{'}\\ z\text{'}\end{array}]=[\begin{array}{ccc}1 & 0 & \lambda \\ 0 & 1 & 0\\ 0 & 0 & 1\end{array}]\,[\begin{array}{c}x\\ y\\ z\end{array}]$$

for *λ* = 0.05. (matrix also shown in Fig. 7C). For images of uniformly rotated networks we applied successive rotations of filaments by *θ* = 3.5° around the *z* axis, according to:

9 $$[\begin{array}{c}x\text{'}\\ y\text{'}\\ z\text{'}\end{array}]=[\begin{array}{ccc}cos\theta  & -sin\theta  & 0\\ sin\theta  & cos\theta  & 0\\ 0 & 0 & 1\end{array}]\,[\begin{array}{c}x\\ y\\ z\end{array}]$$

A synthetic image was generated after each shear or rotation transformation (Figs 7B, 8A).

## Results

We apply our method to three types of biopolymer networks that indicate its broad applicability. The first is the case of polymerizing and intersecting actin filaments along a 2D slide for which we used both simulated^8^ and experimental^10^ images. The second type is ring formation and constriction in mutant fission yeast lacking the contractile ring anchoring precursor component Mid1^23^. The third type is 3D biopolymer networks undergoing shear or simple uniform rotation around an axis. For the latter case we show simulated images and an example of cross-linked fibrin bundles undergoing an externally-imposed mechanical shear deformation. Examples of these images and corresponding TSOAX parameter files can be found in the TSOAX website.

### Tracking Actin Filaments that Intersect during Elongation

As a first test and application of our algorithm, we performed fully automated tracking and measurement of elongation rates in simulated TIRFM images (Fig. 3). In the simulated images, filaments elongated at 12 pixels per frame from one end, similar to actin filaments that elongate much faster from their barbed ends compared to the pointed ends^24^. New filaments were initiated throughout the image sequence, to simulate nucleation of new actin filaments at random location on the image. The images and added noise were constructed by modifying a method in Smith *et al*.^8^.

The intersections that form in the image do not represent physical links, hence we are interested in a network of linear curves without side branches. Our algorithm takes into account the history of filament elongation, which aids in proper linking of extracted curves at junction points.

The program successfully tracked many filaments as they grew past intersection points with other filaments. The blue curves in Fig. 3A show snakes that belong to the same track (three tracks shown). A plot of length versus time for all tracks detected by SOAX is shown in Fig. 3B. Most tracks show the linear growth of 12 pixels/frame in the ground truth data, which can also be identified clearly by a histogram of the length change per frame (Fig. 3C). Tracking errors are seen as tracks with filaments that stop elongating, which correspond to failures to track a filament past an intersection (because of snakes that stopped elongating at a junction point). These events cause a smaller peak around 0 in the histogram of Fig. 3C. Other tracking errors include a sudden change in filament length and premature termination, as seen in Fig. 3B. Overall, the elongation rate was successfully extracted without the need for manual or semi-automated tracking.

We then proceeded to apply our method to experimental time-lapse sequence of fluorescently-labeled polymerizing actin filaments imaged by TIRFM (Fig. 4A). We used the data from Fujiwara *et al*.^10^ for which manual tracing and semi-automated tracking with active contours of selected filaments in Li *et al*.^25^ gave elongation rates of 11.2–11.3 subunits/sec. In the experiments by Fujiwara *et al*.^10^ the filaments grew parallel to a glass slide by polymerization, at higher density and noise compared to Fig. 4. As can be seen in Fig. 4B,C, the measured elongation rate with fully automated detection and tracking has a peak that corresponds to 11.0 subunits/sec after fitting the data with a Gaussian that excludes the peak of the histogram at 0. This is in good agreement with the measurements in Li *et al*.^25^. An advantage of the new method is that it includes data from all filaments on the slide, bypassing any user bias in selecting filaments for their ease for manual or semi-automated tracking.

Finally, as a measure of the accuracy of the tracking algorithm, output tracks for the experimental image in Fig. 4 were manually checked by counting the number of correct correspondences (*TP*, for “true positives”) between snakes in adjacent frames, the total number of correspondence generated (*P*, for “positives”), and total number of true correspondence in the sequence recorded (*TP* + *FN*, where *FN* are “false negatives”). Thus we evaluated the tracking performance to have Precision = *TP*/*P* = 97.9% and Recall = *TP*/(*TP* + *FN*) = 93.1%. These number show a high accuracy of tracking between two successive frames but also indicate a limitation in generating reliable tracks for 10 or more frames for this image series. The local matching procedure is important for this level of accuracy, since without the local matching procedure implemented, the Precision and Recall were calculated to be 80.3% and 73.4%, respectively.

### Tracking Constriction of Tilted Contractile Ring

As an example of an application of our method and software in live cells, we measured the constriction rate of contractile rings in fission yeast cells. A ring can be represented as a closed snake in TSOAX. In wild type fission yeast cells the actomyosin contractile ring forms in the middle of their tubular shape, perpendicular to their long axis. However, in *mid1* Δ cells that lack the ring anchoring protein Mid1, the ring initially assembles in a tilted and non-circular shape^23^. In Fig. 5A, new confocal microscopy images obtained as in Lee *et al*.^23^ for a *mid1* Δ cell expressing Rlc1-GFP (a light chain of type II myosin Myo2p) are shown over time. A non-circular constricting myosin closed loop forms out of linear segments associated with the cell’s cortex. The loop constricts, reorients and becomes circular over time. Our program can be used to detect and track the location of the ring precursor myosin linear elements and track the loop over time through the constriction process (Fig. 5B). The length versus time of tracked segments shows that the loop constricts at a nearly constant rate from the time of ring detection (Fig. 5C, red line). We note that since myosin is not uniformly distributed along the actomyosin ring, the program did not detect a closed loop in frames prior to frame 28 while some low intensity regions are not detected.

### Measuring Nematic Ordering of Strained 3D Fibrin Networks

To illustrate the application of TSOAX to 3D network images, we took time-lapse sequences of a 3D fibrin bundle network. The network was imaged by confocal microscopy with externally-imposed shear deformation in increments of 10% and up to 200% (Fig. 6). The fiber structures in the image are cables of bundled fibrin protofibrils, which establish a physically interconnected 3D network with very few free fiber ends^26^.

Of interest in this experiment are the deformation and aligning of the fibers in response to the macroscopic external perturbation. We used TSOAX to process a sequence of images in time and calculate the change in the nematic order parameter *S*^27,28^, a scalar metric that measures the degree of orientational order. For a completely isotropically orientated assembly of filaments *S* = 0, while *S* = 1 for fully aligned filaments. The graph in Fig. 6 shows the monotonic increase in the nematic order parameter with increasing shear strain, which signifies the alignment of fibrin fibers along the shear direction. The nematic parameter starts from a non-zero value at zero strain, which could indicate alignment due to shear forces during sample preparation as well as the minimum detectable anisotropy due to finite size fluctuations and signal to noise limitations. This analysis provides a direct way to quantify the strain-dependence of alignment, which is an important quantity because it influences the nonlinear elastic response of the network^29,30^.

In Fig. 6, we focused on illustrating the ability of TSOAX to facilitate sequential network extraction, without analysis of individual fiber segment tracks that was at the limits of experimental resolution. Sequential network extraction without fiber-level tracking information is also possible in SOAX^1^, however the workflow is more much more involved as it requires setting up a batch process over a directory of separate 3D images.

### Tracking Simulated 3D Polymer Network Undergoing Shear or Rotation

Mechanical properties of biopolymer networks depend on the deformation and dynamics of individual network segments^31^. To further demonstrate the use of TSOAX in tracking network deformation and dynamics, we created a synthetic movie of a polymer network undegoing shear deformation starting from a Brownian dynamics simulation of attractive semiflexible polymers (Fig. 7A–C). TSOAX was applied without (Fig. 7B) or with snake grouping. The program was able to correctly track the main features of the network. We were able to accurately recover the transformation matrix between the first two frames: the nine elements of matrix *A* in Fig. 7C were determined as follows. Considering that extracted snakes were approximately straight lines (the simulated filaments and filament bundles were much shorter than the single actin filament persistence length), we found the unit vector along the direction defined by the two ends of tracked snakes. This defines column matrices *X* (frame 0) and *X*′ (frame 1) containing the corresponding *x*, *y*, *z* unit vector coordinates in the two frames. Since *X*′ = *AX* gives three equations for the components of *A*, we selected three tracked snakes to get nine equations for the nine coordinates of *A*. We averaged the elements of *A* over 10 such triplets.

Non-affine deformations of sheared polymer networks are predicted to be important in determining their mechanical behavior^31^. TSOAX could be useful in quantifying any deviations from affine deformation under applied force. The transformation of Fig. 7B is affine so we checked how accurately TSOAX can capture it. We measured the angle *ψ* between the axis of a snake detected at frame *k* + 1 and the angle the snake of frame *k* of the same snake track should have after applying the imposed shear transformation (Fig. 7E). Here we assumed each snake can be approximated as a straight line defined by its two ends. In the limit of perfect detection and tracking, the snake rotation between frames *k* and *k* + 1 would be exactly that defined by the affine shear transformation and thus the angle *ψ* would be zero. Non-zero values of *ψ* correspond to incorrect detection of non-affine deformations. We found that most such *ψ* angles are less than 5° (Fig. 7F,G). This illustrates that non-affine deformations larger than this angle may be detectable by TSOAX (for the conditions of this example). The few events with large angles in the histogram of Fig. 7F,G correspond to tracking errors, typically as a result of differences in topology and junction detection between successive frames. These errors were less frequent with grouping turned on (Fig. 7F,G).

We also applied successive rotations of filaments by 3.5°  around the *z* axis and tracked the rotated network with TSOAX (Fig. 8A). For this transformation, the azimuthal angle of a line along each snake segment *ϕ* (measured as in Xu *et al*.^1^) should increase by 3.5°  per frame while the polar angle *θ* should remain the same. The change of *ϕ* and *θ* of the detected snake tracks are shown in Fig. 8B. The average value agrees well with the imposed rotation, to within fluctuations due to segmentation and tracking errors.

As a measure of tracking accuracy in the 3D images we calculated the Precision, *TP*/*P*, in Fig. 8. Here *TP* are the correct correspondences between snakes in adjacent frames over the total number of correspondences generated, *P*. Considering as correct those correspondences with a change of azimuthal angle within 7°  of the anticipated 3.5°  per frame, we find *TP*/*P* = 75%. This Precision value indicates how the average rotation can indeed be extracted by averaging over several snakes. This value also shows that errors in individual tracks would accumulate after several frames, at a faster rate compared to the 2D images of Fig. 4 for which the Precision was larger. This effect is anticipated given the additional complexities in 3D. Similarly, we estimate the Recall, =*TP*/(*TP* + *FN*) = 42%, is lower compared to the 2D images, accounting as false negatives those detected segments that did not have a correspondence in the subsequent frame.

## Discussion

Application of our network tracking method to experimental images shows its use in a big range of 2D and 3D networks. We showed example of sequences up to 60 frames, containing 1–200 detected snakes per frame. For such image stacks, the time required for TSOAX to establish snake correspondence is typically less than the snake detection time. For images with more snakes per frame and with more frames, the time to establish snake correspondence becomes longer than the snake detection time. To speed up the snake detection step, a smaller density of snake points could be used (such as 1 point every 3 pixels) as well as a reduced minimum required number of iterations per snake evolution (e.g. reducing parameter “Maximum iterations” to 100). Even without the tracking functionality, the program is useful in network detection over multiple time frames (Fig. 6). The data of filament and junction coordinates and tracking correspondence are saved as a text file to enable analysis depending on the particular application.

The accuracy of the tracking results is limited by both the accuracy with which the network of snakes is extracted in every frame and by the accuracy with which correspondence along frames is established. It was shown in Xu *et al*.^1^ that a value of a signal to noise ratio (defined in the local neighborhood of snake point^1^) of order 5 is needed for reliable automated extraction in SOAX, after using a procedure to optimize the parameters based on an optimization function. For images with relatively low signal to noise ratio, there is a delicate balance when tuning the SOAX parameters “Ridge Threshold” *τ* and “Stretch Factor” *k*_*str*_, which control how many snakes are initialized and by how much snakes elongate^1^. This choice may lead to under-detection or over-detection of snakes in the background, some of which can be seen in Fig. 4, for example. Since TSOAX uses the SOAX alogorithm for network extraction for each time frame, the same signal to noise ratio limitation and procedure to optimize parameters also applies in TSOAX. We described how tracking snakes in time introduces additional complexities that may limit track accuracy in ways that vary depending on the particular application.

We illustrated different ways by which the accuracy of the network tracking can be evaluated. For the case of actin filament elongation, this included comparison to simulated time-lapse images with added noise and comparison to results of manual tracking. For deforming networks, we imposed a known affine transformation to an initial image generated by a simulation. A similar procedure of applying a transformation to an initial experimental image can be used to evaluate tracking accuracy. These analyses show a probability of tracking error per frame, per snake, in the range 2–25% but note that this number depends strongly on the type of network in consideration.

Software developed in prior studies to track gliding filaments for *in vitro* motility assays used skeletonization to extract linear filaments in each frame^32^. Tracks of gliding filaments were constructed by connecting filaments between successive frames based on distance criteria (similar to Eq. 3) in addition to criteria that favor gliding along the longitudinal direction (not included in TSOAX). Crossing filaments that occur during gliding filament collisions were excluded from the analysis. TSOAX may be applicable to such systems while also able to track filaments during collisions.

The method and associated TSOAX software should be applicable to a broad class of systems beyond those presented in this paper. This includes images of mitochondrial networks (for which software exists for static images^33^), images of amyloid and cellulose fibers^34,35^, plant cell wall deformation imaged by by AFM^36^, and images of carbon nanotube networks^37^. It may also form a basis in future work to track more complex events such as filament merging, splitting, and overlapping.
